# Unbiased PCR-free spatio-temporal mapping of the mtDNA mutation spectrum reveals brain region-specific responses to replication instability

**DOI:** 10.1186/s12915-020-00890-5

**Published:** 2020-10-23

**Authors:** Emilie Kristine Bagge, Noriko Fujimori-Tonou, Mie Kubota-Sakashita, Takaoki Kasahara, Tadafumi Kato

**Affiliations:** 1 Laboratory for Molecular Dynamics of Mental Disorders, RIKEN Center for Brain Science, Wako, Saitama Japan; 2 Current address: Support Unit for Bio-Material Analysis, Research Resources Division, RIKEN Center for Brain Science, Wako, Saitama Japan; 3 Current address: Career Development Program, RIKEN Center for Brain Science, Wako, Saitama Japan; 4 Department of Psychiatry and Behavioral Science, Juntendo University, Graduate School of Medicine, Hongo 2-1-1, Bunkyo, Tokyo 113-8421, Japan

**Keywords:** Mitochondrial DNA, Polymerase gamma, Polg, Ageing, Mitochondrion, mtDNA mutation, Paraventricular thalamic nucleus, Nucleus accumbens, Substantia nigra, Dorsal raphe

## Abstract

**Background:**

The accumulation of mtDNA mutations in different tissues from various mouse models has been widely studied especially in the context of mtDNA mutation-driven ageing but has been confounded by the inherent limitations of the most widely used approaches. By implementing a method to sequence mtDNA without PCR amplification prior to library preparation, we map the full unbiased mtDNA mutation spectrum across six distinct brain regions from mice.

**Results:**

We demonstrate that ageing-induced levels of mtDNA mutations (single nucleotide variants and deletions) reach stable levels at 50 weeks of age but can be further elevated specifically in the cortex, nucleus accumbens (NAc), and paraventricular thalamic nucleus (PVT) by expression of a proof-reading-deficient mitochondrial DNA polymerase, *Polg*^*D181A*^. The increase in single nucleotide variants increases the fraction of shared SNVs as well as their frequency, while characteristics of deletions remain largely unaffected. In addition, *Polg*^*D181A*^ also induces an ageing-dependent accumulation of non-coding control-region multimers in NAc and PVT, a feature that appears almost non-existent in wild-type mice.

**Conclusions:**

Our data provide a novel view of the spatio-temporal accumulation of mtDNA mutations using very limited tissue input. The differential response of brain regions to a state of replication instability provides insight into a possible heterogenic mitochondrial landscape across the brain that may be involved in the ageing phenotype and mitochondria-associated disorders.

## Background

Ageing is characterised by diverse molecular, physiological, and behavioural changes, the mechanistic onset of which is poorly understood. Time-dependent accumulation of mitochondrial DNA (mtDNA) mutations through a vicious cycle of reactive oxygen species (ROS) production and oxidative damage to mtDNA has been proposed as a possible driver [1], causing ageing-induced mitochondrial dysfunction. However, accumulating evidence suggests that mtDNA is not associated with oxidative damage [2, 3], despite the presence of other oxidative damage in mitochondria [4, 5]. Though it has been highly debated whether mtDNA mutations are cause or effect of ageing [3], mtDNA mutations alone are adequate to drive ageing as seen in the mutator mouse, an ageing model expressing a proof-reading-deficient mitochondrial DNA polymerase *Polg*, *Polg*^*D257A*^ [6]. Evidence suggests the involvement of specific mtDNA mutations in oxidative stress [7, 8], indicating that the 37 genes encoded by mtDNA (13 proteins, 22 tRNAs, and 2 rRNAs) as well as the non-coding control region (NCR) have different contributions to mitochondrial dysfunction. As the NCR interacts with the inner mitochondrial membrane [9] and several proteins [10–12] to form the nucleoid structure and regulate transcription and replication, mitochondria may be highly dependent on the NCR for proper function. What remains unclear is the exact spatio-temporal accumulation of mtDNA mutations between individual organs and even more importantly, the heterogeneity with which mtDNA mutations may accumulate across an organ.

We have developed a variation of the mutator mouse that expresses an alternative proof-reading-deficient *Polg*, *Polg*^*D181A*^, under the control of the CaMKIIα-promoter, resulting in forebrain neuron-specific expression of the transgene [13]. This model can be used to examine the neuron-specific mitochondrial response to replication instability arising from lack of proof-reading of *Polg*, and we have previously demonstrated the accumulation of dysfunctional mitochondria in these mice [14, 15].

Up until now, purified mitochondria from whole organs [16] or some form of either partial or nearly full-length PCR amplification of mtDNA [17–19] have been required for next-generation sequencing. Especially in highly heterogeneous tissue such as the brain, the cellular composition, metabolic profile, and local environment could potentially harbour regional mutational *hotspots* that may contribute to both the ageing phenotype and various disorders, but such limited tissue regions have been too small to study without PCR amplification. PCR amplification not only introduces a bias to the study of mtDNA, it also makes it difficult to identify rearrangements of mtDNA such as deletions and duplications unless full-length amplification of mtDNA is performed.

In this study, we implemented a method to prepare mtDNA for next-generation sequencing without PCR amplification prior to library preparation using DNA extracted from small brain dissections from mice during ageing. We show that mice accumulate single nucleotide variants (SNVs) and deletions with ageing across all brain regions in a largely homogenous manner. However, the expression of *Polg*^*D181A*^ causes a highly brain region-specific increase in the ageing-induced accumulation of SNVs and deletions. Our data demonstrate a previously undescribed bimodal distribution of deletion sizes across both genotypes. In addition, we demonstrate a *Polg*^*D181A*^-dependent and ageing-induced accumulation of NCR-containing multimers. In all, our unbiased approach to map the full spatio-temporal mtDNA mutation spectrum provides an unprecedented method to gain insight into brain-wide mitochondrial heterogeneity.

## Results

### Implementation of a method for isolation of mtDNA from small tissue samples

**Fig. 1:**
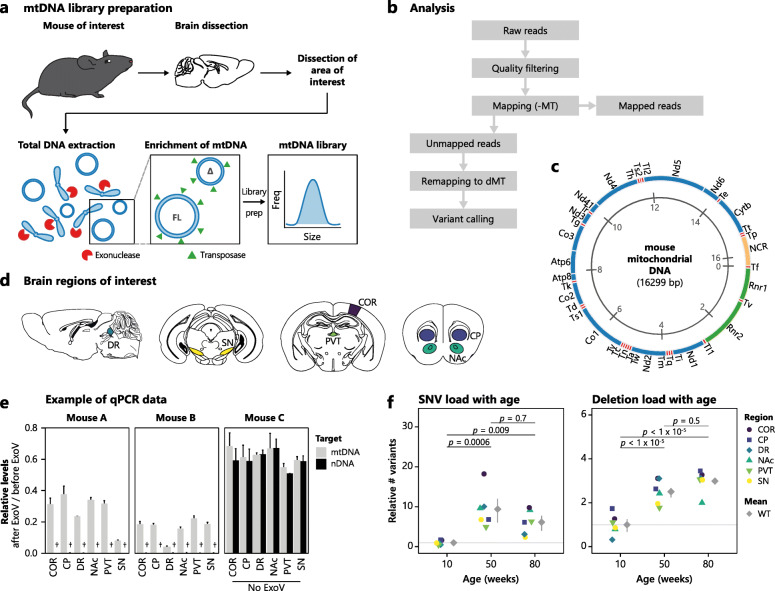
Ageing increases the load of both SNVs and deletions in mtDNA across all brain regions. **a** Schematic illustration of the workflow from mouse to prepared library. Briefly, brain regions of interest were rapidly sampled and total DNA was extracted. Linear DNA was enzymatically degraded by exonuclease (ExoV), and non-linear DNA is purified and used for library preparation. FL: full-length mtDNA molecule, ∆: mtDNA molecule with deletion. **b** Overview of the analysis workflow to optimise mtDNA variant detection. Shortly, after quality filtering, reads were mapped to mm10 without the mitochondrial chromosome (MT). Unmapped reads were then re-mapped to a modified MT reference (dMT: two MT references in tandem) and variants called. **c** Overview of mouse mtDNA. Green: rRNA encoding genes; blue: protein-coding genes; red: tRNA-encoding genes; orange: non-coding region (NCR). **d** Schematic showing the areas isolated as the cortex (COR), caudate putamen (CP), dorsal raphe (DR), nucleus accumbens (NAc), paraventricular nucleus of the thalamus (PVT), and substantia nigra (SN). **e** DNA stored before and after ExoV digestion was subjected to qPCR to determine the relative levels of three mtDNA and three nuclear targets before and after digestion (shown for two different mice, A and B). Mouse C was treated as A and B but without the addition of ExoV. Bars show the mean of target signals and the standard deviation is indicated. †: nDNA after ExoV treatment was not detected or only detected at a very low level by qPCR and may not be visible in the bar plot. **f** Dot plot illustrating the age-dependent increase in the load of SNVs (left) and deletions (right) across the investigated brain regions (as indicated by the colour legend). All samples have been normalised to the mean of the variants at 10 weeks. Grey diamonds indicate the mean of all regions at the indicated age, and the 95% confidence interval is shown. Three-way ANOVA showed age, not region or animal, significantly (*p* < 0.01) contributed to SNV and deletion levels. Tukey’s test was used *post hoc* to determine *p* values between each age group

To investigate the full mtDNA mutation spectrum in small mouse brain regions and avoid the inherent bias present in PCR amplification, we implemented a method for the enzymatic depletion of nuclear DNA (nDNA) from total DNA [20] extracted from brain tissue. We enzymatically depleted nDNA from total DNA by treatment with exonuclease V, an enzyme that targets the free ends of linear DNA essentially leaving circular mtDNA intact [20]. We used mtDNA-enriched samples directly for library preparation for next-generation sequencing (Fig. 1a).

We allowed for split-read mapping to identify deletions with BBMap [21] (Fig. 1b) using a custom mtDNA reference composed of two mm10 MT references in tandem (dMT). In a two-round mapping approach, we removed residual nDNA-derived sequencing reads, especially due to the presence of nuclear mitochondrial DNA segments (*Numts*), i.e. mtDNA-like sequences in the nuclear genome. Due to the circularity of mtDNA (Fig. 1c), deletions may span the “ends” of the mtDNA reference, that is linear in nature, which will interfere with deletion calling. By using dMT, we circumvented this and were able to reliably detect variants at any position in mtDNA. As sequencing reads generated by Nextera are well known to exhibit GC bias in the first bases of the read, we trimmed these bases and excluded an additional 5 bp at the read ends during variant calling (see the “Methods” section). Based on this, we have no reason to believe that variant calling is influenced by transposase sequence bias. For identification of mtDNA variants, we sampled the sensory cortex (COR), caudate putamen (CP), dorsal raphe (DR), nucleus accumbens (NAc), paraventricular thalamic nucleus (PVT), and substantia nigra (SN) (Fig. 1d) during mouse ageing and confirmed nDNA depletion by qPCR before library prep and sequencing (Fig. 1e).

### Ageing-related accumulation of SNVs and deletions across all brain regions

We initially mapped the ageing-related changes in mtDNA mutations across the brains of 10-, 50-, and 80-week-old wild-type (WT) mice (Fig. 1f). As expected, the number of SNVs in 10-week-old mice was very low but rose on average 10-fold in 50-week-old mice and remained relatively unchanged at 80 weeks (Fig. 1f, left). Similarly, deletions also reached a plateau at 50 weeks after increasing 2.5–3-fold from 10 weeks (Fig. 1f, right). The plateau reached in both SNVs and deletions at 50 weeks indicates a restriction in the load of mtDNA mutations. This may be imposed by loss of mitochondria function, thus limiting its propagation or triggering mitophagy. Alternatively, selective replication of mtDNA molecules may keep the mutation load from further increasing. In all, both SNVs and deletions appeared homogenously accumulated across the examined brain regions during ageing, but may be influenced by different pathological settings.

### *Polg*^*D181A*^ expression causes brain region-specific SNV accumulation with ageing

**Fig. 2:**
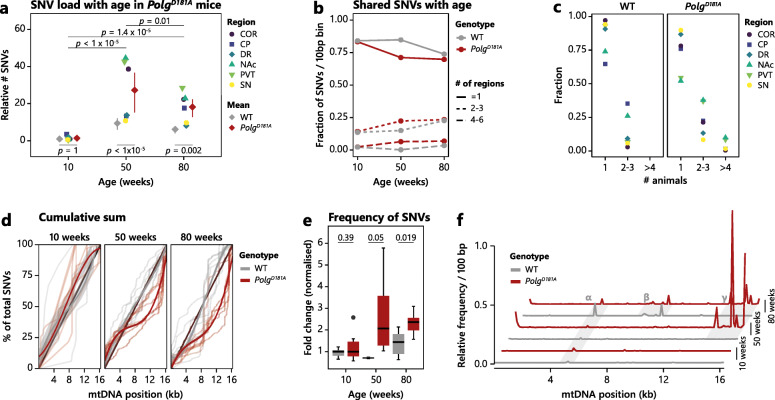
SNVs heterogeneously accumulate across brain regions in *Polg*^*D181A*^ mice and cause mtDNA position-specific mutational patterns. **a** Dot plot illustrating the age-dependent increase in the load of SNVs in *Polg*^*D181A*^ mice across the investigated brain regions (as indicated by the colour legend) normalised to the mean of WT samples at 10 weeks. Grey diamonds indicate the mean of WT-derived brain region samples for reference (same as in Fig. 1f). Red diamonds indicate the mean of *Polg*^*D181A*^-derived brain region samples and the 95% confidence interval is shown. Three-way ANOVA (age, region, and animal) of *Polg*^*D181A*^-derived samples showed that age significantly contributed to SNV levels (*p* values of *post hoc* Tukey’s test are shown). Three-way ANOVA showed a significant contribution of all variables (age, genotype, region). *p* values of *post hoc* Tukey’s test comparing WT and *Polg*^*D181A*^ at each age are shown. For region contribution, we found a significant contribution of COR, NAc, and PVT to SNV levels in *Polg*^*D181A*^ mice using a linear model for main effects. **b** SNVs were counted in 10-bp non-overlapping bins for WT (grey) and *Polg*^*D181A*^ (red) mice at 10, 50, and 80 weeks, and the number of regions with SNV in each bin calculated. Note that in the case that one region has more than one SNV in a bin, it is only counted as one instance of an SNV. The overlap was visualised for non-overlapping bins (“1”), bins shared across two or three regions (“2–3”), and bins shared across four to six regions (“4–6”). **c** SNVs were counted in 10-bp non-overlapping bins for WT (grey) and *Polg*^*D181A*^ (red) mice at 50 weeks, and the number of individual animals with SNVs in each bin calculated. Note that in the case that one animal has more than one SNV in a bin, it is only counted as one instance of an SNV. The overlap was visualised for non-overlapping bins (“1”), bins shared across two or three animals (“2–3”), and bins shared by four or more animals (“≥ 4”). **d** Cumulative percentage of SNVs detected in each examined brain region (thin lines) for both WT (grey) and *Polg*^*D181A*^ (red) at 10, 50, and 80 weeks old. Bold lines indicate the smooth conditional mean for each genotype. **e** The relative average SNV allele frequency for each region for WT (grey) and *Polg*^*D181A*^ (red) mice at 10, 50, and 80 weeks as indicated shown as boxplots. *p* values of two-sided *t* tests are shown. **f** SNVs across brain regions were pooled for each genotype at each age and divided into 100-bp bins across the mtDNA reference and the allele fraction for SNVs in each bin summed and normalised (i.e. highest peak set to 1). Grey areas indicate mtDNA regions where peaks are found across all variables (*α*), peaks that are ageing-dependent (*β*), and ageing-induced *Polg*^*D181A*^-dependent peaks (*γ*)

Having established that our method could be used to map ageing-induced mtDNA mutations, we turned to our *Polg*^*D181A*^ model mice to investigate the brain region-specific influence of proof-reading deficiency. We sequenced mtDNA from the six brain regions of interest from *Polg*^*D181A*^ mice and identified the SNVs in each region at 10, 50, and 80 weeks of age (Fig. 2a).

In young mice, there was no change in SNV levels between WT and *Polg*^*D181A*^ in 10-week-old mice. At 50 and 80 weeks, we observed a significant increase in SNV levels which was especially prominent in COR, NAc, and PVT (Fig. 2a). This demonstrated that the heterogeneity of the mitochondrial response to proof-reading deficiency is present across an organ and not only between organs [6, 16, 22, 23].

Importantly, we found no relationship between the expression of the *Polg*^*D181A*^ transgene in the investigated brain regions and the level of detected SNVs (Additional file 1: Fig. S5c).

### SNVs are excessively shared between brain regions

We wondered whether the increase in SNVs with both ageing and *Polg*^*D181A*^ expression was affecting the same positions in mtDNA across brain regions. Indeed, looking in 10-bp non-overlapping intervals, we found an increase in shared SNV positions with ageing which was enhanced by *Polg*^*D181A*^ expression (Fig. 2b). The overlap between individual animals was most prominent in PVT and NAc (Fig. 2c). As *Polg*^*D181A*^ expression introduced a shift of the SNV distribution to the right side of the distribution plot (i.e. towards the NCR) compared to WT (Fig. 2d), we examined where shared SNVs were located. We found that shared SNV positions were significantly different from non-shared SNV positions in *Polg*^*D181A*^ mice (*t* test, *p* < 1 × 10^−5^) but not in WT (*t* test, *p* = 0.254) when looking across all ages and shifted towards the 3′ region (i.e. towards the NCR) (Additional file 1: Fig. S1a).

The increase in shared SNVs was accompanied by a significant increase in SNV frequency with *Polg*^*D181A*^ expression (Fig. 2e) which was driven by high frequency SNVs in specific mtDNA regions (Fig. 2f). These regions appeared highly context-dependent, i.e. peaks that are found across all samples (“α” on Fig. 2f), only in very aged mice (“β”), or are *Polg*^*D181A*^-specific (“γ”). This was mimicked in the *Pearson* correlation, where most brain regions from 50- and 80-week-old *Polg*^*D181A*^ mice form a distinct cluster and most samples from 10-week-old mice form a distinct cluster (Additional file 1: Fig. S1b) and we found specific SNV *hotspots* in 10-week-old animals independent of genotype (Additional file 1: Fig. S1c). In addition, there was a significant overlap of the specific positions at which SNVs are present in COR, NAc, and PVT (the brain regions most sensitive to *Polg*^*D181A*^ expression) at both 50 and 80 weeks in *Polg*^*D181A*^ mice (Additional file 1: Fig. S1d, right). For WT mice, the overlap is only pronounced at 80 weeks and *p* values do not reach similar levels of significance (Additional file 1: Fig. S1d, left).

We found an increase of SNVs in the NCR and complex III genes (Additional file 1: Fig. S1e), while transitions and transversions (Additional file 1: Fig. S1f) were comparable to those of previous studies, and we saw no indication of either ageing- or *Polg*^*D181A*^-induced oxidative mutations [19, 24], together with no change in the types of mutations (Additional file 1: Fig. S1g).

Together, these data demonstrated a brain region-specific ageing-dependent *Polg*^*D181A*^-induced mtDNA SNV spectrum, where COR, NAc, and PVT are regional *hotspots*. In addition, certain mtDNA positions are highly sensitive to SNVs and seem to function as context-dependent mutational *hotspots*.

### Ageing-dependent *Polg*^*D181A*^-induced deletions accumulate in the same brain regions as SNVs

**Fig. 3:**
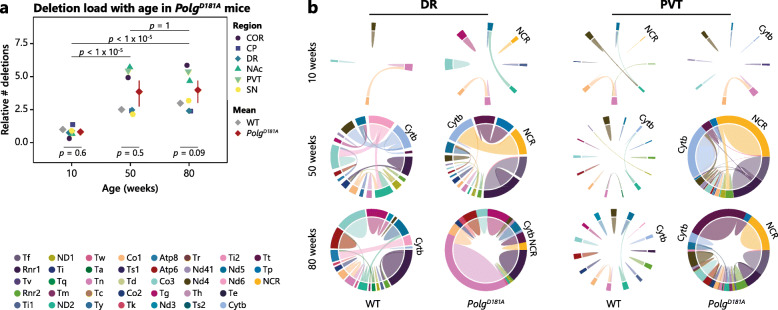
Accumulation of deletions induced by *Polg*^*D181A*^ expression are brain region-specific and ageing-dependent. **a** Dot plot illustrating the age-dependent increase in the load of SNVs in *Polg*^*D181A*^ mice across the investigated brain regions (as indicated by the colour legend) normalised to the mean of WT samples at 10 weeks. Grey diamonds indicate the mean of WT-derived brain region samples for reference (same as in Fig. 1f). Red diamonds indicate the mean of *Polg*^*D181A*^-derived brain region samples and the 95% confidence interval is shown. Three-way ANOVA (age, region, and animal) of *Polg*^*D181A*^-derived samples showed that age significantly contributed to deletion levels (*p* values of *post hoc* Tukey’s test are shown). *p* values of three-way ANOVA (age, genotype, region) with *post hoc* Tukey’s test are shown for each age group. For region contribution, we found a significant contribution of COR, NAc, and PVT to deletion levels in *Polg*^*D181A*^ mice using a linear model for main effects. **b** Chord diagrams indicating the deletions accumulated at 10, 50, and 80 weeks in DR and PVT from WT and *Polg*^*D181A*^ mice. Data is normalised pr. brain region, and the width of each gene indicates the summed allele fraction of deletions spanning the indicated gene(s). The colour of the chord indicates the gene in which the breakpoint 5′ position is located. Plots were made using *circlize*

We next turned our attention to the influence of *Polg*^*D181A*^ expression on the accumulation of deletions. We found a significant ageing-induced accumulation of deletions in both 50- and 80- compared to 10-week-old *Polg*^*D181A*^ mice, but we observed no significant differences between WT and *Polg*^*D181A*^ at any age (Fig. 3a). However, deletion accumulation in response to *Polg*^*D181A*^ showed a prominent region specificity. While CP, DR, and SN *Polg*^*D181A*^ SNV levels were only slightly elevated compared to WT mice, COR, NAc, and PVT showed a very high accumulation of deletions at 50 and 80 weeks. We found a significant difference in deletion levels between these regions compared to the other regions in *Polg*^*D181A*^ when pooling data from 50- and 80-week-old mice (*p* = 0.002, one-way ANOVA). Similar to SNVs, we found no indication that expression levels of the *Polg*^*D181A*^ transgene were the major driver of deletion levels in the *Polg*^*D181A*^ mice (Additional file 1: Fig. S5c).

The differences across brain regions with ageing of WT and *Polg*^*D181A*^ mice can be appreciated by chord diagrams showing the span of all deletions at each time point (Fig. 3b). Where PVT showed both an ageing-induced and a clear ageing-dependent *Polg*^*D181A*^-induced accumulation of deletions, DR only showed an ageing-induced accumulation of deletions, highlighting the brain region-specific mtDNA sensitivity to a setting of replication instability. *Pearson* correlation indicated similarities in the deletions found with ageing of *Polg*^*D181A*^ mice (Additional file 1: Fig. S2a), indicating that *Polg*^*D181A*^ expression induces a specific landscape of mtDNA deletions.

### Deletions share characteristics independent of genotype

**Fig. 4:**
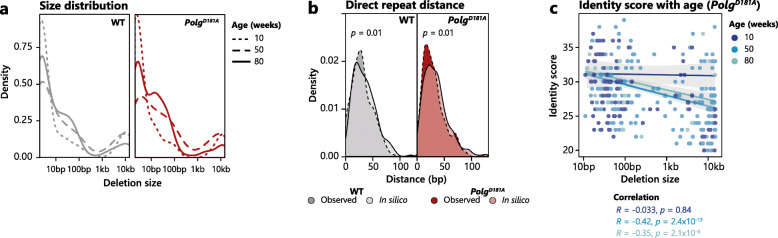
Characteristics of deletions change with age, but not the expression of *Polg*^*D181A*^. **a** Density plot of deletion sizes for WT (grey) and *Polg*^*D181A*^ (red) for 10- (dotted line), 50- (dashed line), and 80-week-old (full line) mice. **b** The shortest average distance from 5′ and 3′ deletion breakpoint pairs to a direct repeat pair in the mitochondrial genome for the observed deletions (darker colour) and a random in silico generated deletion length-matched library (lighter colour) for both WT (left, in grey) and *Polg*^*D181A*^ (right, in red) using pooled data from all ages and brain regions examined for each genotype. *p* values of two-sided *t* tests are shown. **c** *Needle* identity score calculated in a ± 10 bp window at the 5′ and 3′ deletion breakpoints as a function of deletion size after pooling of WT and *Polg*^*D181A*^ samples. Correlation for each age is indicated by the full lines and correlation data indicated in the same colour code

The positions at which deletions start and end are termed breakpoints and based on the co-occurring deletions between brain regions from *Polg*^*D181A*^ mice, we hypothesised that breakpoints must be shared between different samples. We looked in 100-bp bins along the mtDNA and found that shared breakpoints cluster in very distinct locations (Additional file 1: Fig. S2b). Some shared breakpoints are age- and genotype-independent (~ 5 kb) whereas others are genotype-dependent (~ 15 kb). Sizes of deletions themselves follow a bimodal distribution independent of age and genotype and can be roughly divided into those < 100 bp and those > 1 kb, with few observations in the intermediate range (Fig. 4a). Even though the number of deletions in 10-week-old animals is low, they still follow this distribution, though the fraction of very small deletions is high compared to aged animals.

### Molecular determinants of deletions

A previous study has suggested that the majority of mtDNA deletions in Parkinson’s patients occur at direct repeats [17], a proposed [25] though highly debated [26] feature of human mtDNA deletions. To investigate the influence of direct repeats in breakpoint formation in the mouse brain, we identified direct repeats ≥ 8 bp in mtDNA (Additional file 1: Fig. S2c). After pooling deletions per genotype, we identified the direct repeat pair with the shortest average distance from the 5′ and 3′ breakpoints of WT and *Polg*^*D181A*^ deletions as well as for in silico generated, deletion length-matched deletion libraries for each genotype (see the “Methods” section). The shortest average distance was shorter for experimentally derived deletions than randomly generated deletions for both WT and *Polg*^*D181A*^ mice (Fig. 4b), but there was no difference between WT and *Polg*^*D181A*^ (*t* test, *p* = 0.905). This indicates that direct repeats may contribute to at least a part of the identified deletions. We found this to be the case at 10 and 50 weeks but not 80 weeks (Additional file 1: Fig. S2d), as deletions are significantly closer to direct repeats than the in silico deletion libraries for both WT and *Polg*^*D181A*^ mice.

Restriction of sequence similarity to direct repeats is a rigorous criterion. We therefore calculated the sequence identity scores in a 20-bp window surrounding all 5′ and 3′ breakpoints (i.e. 10 bp on each side of the breakpoint). We found a negative correlation between sequence identity score and deletion length in 50- and 80-week-old *Polg*^*D181A*^ mice (Fig. 4c) and further saw a significant difference between the identity scores of deletions < 100 bp and > 100 bp at 50- and 80-week-old *Polg*^*D181A*^ as well as WT mice (Additional file 1: Fig. S2e). These data imply a differential contribution of non-direct repeat sequence similarity to short and long deletions.

### Abundant NCR multimers are exclusive to *Polg*^*D181A*^-expression

**Fig. 5:**
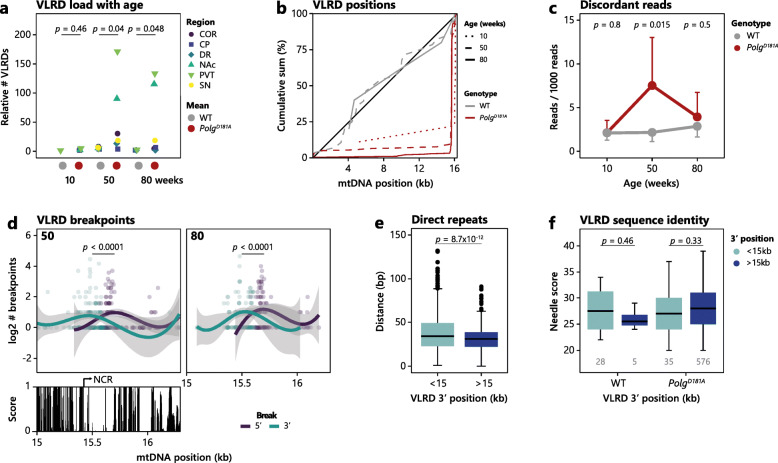
Putative NCR multimers are *Polg*^*D181A*^-specific and accumulate with age in a highly brain region-specific manner. **a** Dot plot illustrating the age-dependent increase in the load of VLRDs in *Polg*^*D181A*^ mice across the investigated brain regions (as indicated by the colour legend). All samples have been normalised to the sample with the lowest number of detected variants. **b** Cumulative percentage of 5′ position of VLRDs (i.e. start position of the putative multimeric sequence) summed across brain regions for WT (grey) and *Polg*^*D181A*^ (red) for 10- (dotted line), 50- (dashed line), and 80-week-old (full line) mice. **c** Mean number of discordant reads as extracted by *samtools* at 10, 50, and 80 weeks for WT (grey) and *Polg*^*D181A*^ (red) and standard deviation is indicated. *p* values of two-sided *t* tests are shown. **d** Summed analysis of mtDNA breakpoints of VLRDs from *Polg*^*D181A*^ mice in the NCR and surrounding region. 5′ (purple) and 3′ (dark turquoise) breakpoints are summed at each position across all brain regions at either 50 (left) or 80 (right) weeks old, and smooth conditional means are plotted. The lower panel shows the *phastCons* conservation score via the UCSC genome browser in the same region. *p* values of two-sided *t* tests are shown. **e** Boxplot showing the shortest average distance to a direct repeat of all *Polg*^*D181A*^ VLRDs separated by VLRD 5′ position into **<** 15 kb (light blue) or **>** 15 kb (dark blue). *p* values of two-sided *t* tests are shown. **f** Boxplot showing the *Needle* identity score of WT and *Polg*^*D181A*^-derived VLRDs pooled across ages and brain regions examined split into VLRDs with 3′ position < 15 kb (light blue) and > 15 kb (dark blue). *p* values of two-sided *Wilcoxon* tests are shown

As expected, the sequencing coverage exhibited some variability, likely associated with a slight sequence specificity of the transposase used for library preparation [27–29]. However, in specific brain regions from the 50- and 80-week-old *Polg*^*D181A*^ mice, we observed an increased coverage in the 15 kb+ region including at least a part of the NCR (Additional file 1: Fig. S3a). Localised increased coverage is often thought to be associated with duplicated regions. As mitochondrial DNA is circular, it is not possible to distinguish small duplications from very long-range deletions (VLRDs) (Additional file 1: Fig. S3b). We therefore wondered if our data of mtDNA deletions could support the presence of multimers. By classifying VLRDs as deletions > 15 kb, we found that VLRDs are specifically enriched in NAc and PVT from 50- and 80-week-old *Polg*^*D181A*^ mice (Fig. 5a) and enriched in the 15 kb+ region (Fig. 5b and Additional file 1: Fig. S3c), supporting the idea that mtDNA multimers including at least part of the NCR accumulate in a brain region-specific and *Polg*^*D181A*^-dependent manner with age. We also found an increase in discordant reads in 50-week-old *Polg*^*D181A*^ mice, which further supports the presence of genomic rearrangements such as multimers (Fig. 5c and Additional file 1: Fig. S3c).

These putative multimers appear to form in a quite restricted region of mtDNA as their 5′ and 3′ “breakpoints” (indicating the end and the start of the duplicated sequence, respectively), accumulate at rather discrete positions (Fig. 5d) spanning a region with a low conservation score across mammals (Fig. 5d, bottom panel). Previous data suggested the presence of multimers in the brain from the mutator mouse [16]. We used the same PCR approach as Williams et al. and validated the *Polg*^*D181A*^-specific presence of multimers (Additional file 1: Fig. S4a, top and middle panel). An alternative PCR setup that would only yield a product in the presence of multimers confirmed these results (Additional file 1: Fig. S4a, bottom panel) and subsequent data also indicated the presence of inversions (Additional file 1: Figs. S4b,c).

Together, these data support the presence of highly brain region-specific ageing-induced *Polg*^*D181A*^-dependent multimers which are highly specific to a partial NCR-containing segment of mtDNA specifically in NAc and PVT.

### Direct repeats may be involved in NCR multimer formation

Multimers can be formed by several mechanisms. One mechanism is by strand slipping during replication which may be influenced by the local environment surrounding the NCR, which is known to interact with the inner mitochondrial membrane [9]. Another mechanism is mediated by the DNA sequence surrounding the start and end positions of the multimer region. In support of the idea of strand slipping, we find VLRDs in the 15 kb+ region to be closer to direct repeats compared to multimers in other parts of the mtDNA (Fig. 5e), though the overall sequence similarity surrounding breakpoints is not different (Fig. 5f). SNVs were enriched near VLRD 5′ breakpoints as well as ~ 7 kb upstream with a mean distance of 75  ± 462 bp to the nearest SNV (Additional file 1: Fig. S5a). Fifteen percent of VLRD breakpoints co-position with SNVs, a number which is not influenced by discordant reads. SNVs were not enriched within the putative multimeric region (Additional file 1: Fig. S5b).

### Transgene expression level does not drive variants

The expression of transgenes are often not similar across tissues, which is also true for *Polg*^*D181A*^ expression [14]. To confirm that the expression differences were not driving the differences we observed in the accumulation of mutations in response to *Polg*^*D181A*^ expression, we evaluated the expression levels of endogenous *Polg* and transgenic *Polg*^*D181A*^. Importantly, we were interested in the relative expression levels of the two transcripts, as endogenous and transgenic *Polg* will be competing for access to mtDNA during replication. As presented in previous sections, we find no correlation between mtDNA mutation levels and relative *Polg*^*D181A*^/*Polg* levels at any age (Additional file 1: Fig. S5c). Together, this demonstrates that transgene expression levels were not the major driver of brain region specificity to proof-reading deficiency in mitochondria.

### mtDNA variants cluster together along genomic regions

**Fig. 6:**
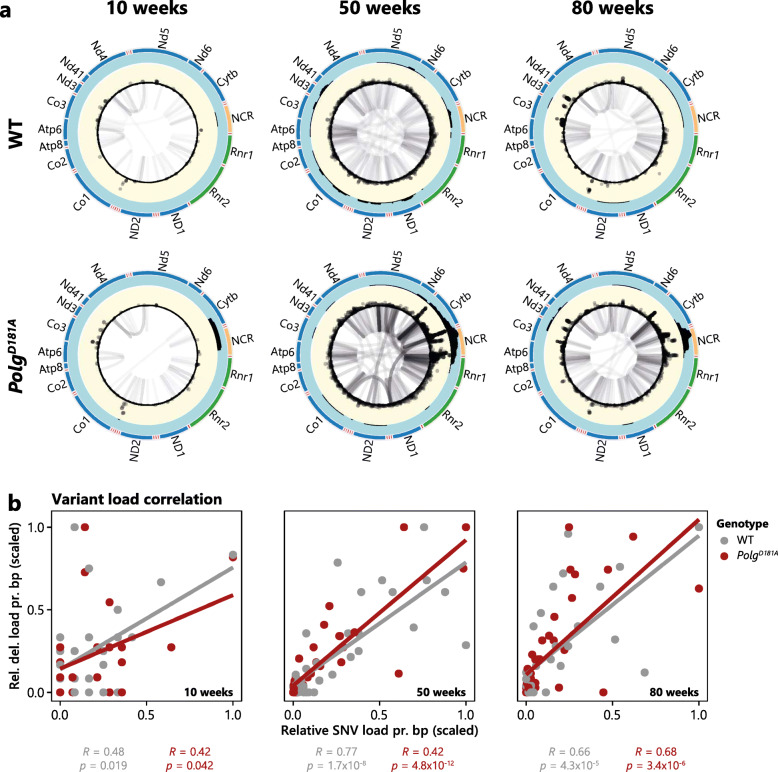
Levels of different variants correlate across mtDNA genes. **a** Identified variants plotted across the mtDNA reference for WT (top) and *Polg*^*D181A*^ (bottom) samples across all regions at 10, 50, and 80 weeks as indicated. Tracks from outside to inside: (1) mtDNA gene names (note that tRNA gene names are not shown), (2) mtDNA genes by length, (3) the relative level of VLRDs (e.g. multimers and inversions), (4) SNVs detected across all regions with height indicating log2-transformed allele frequency, and (5) deletions plotted as lines connected to start and end positions. Note that the start and end points of deletions are not indicated. **b** Load of SNV and deletion for each mtDNA gene divided by the gene length are plotted for WT (grey) and *Polg*^*D181A*^ (red) for 10-, 50-, and 80-week-old mice. Data was scaled (from 0 to 1) before plotting for clarity. *Pearson* correlation and significance of the correlation is shown below each plot for both WT (grey) and *Polg*^*D181A*^ (red)

Throughout our analysis of the mutation spectrum of mtDNA from both WT and *Polg*^*D181A*^ mice, it became increasingly clear that different types of variants often were found in specific mtDNA regions. To further investigate this, we plotted all variants analysed—SNVs, deletions, VLRDs (i.e. multimers)—across mtDNA in a circular plot (Fig. 6a). Visual inspection of this plot showed that different types of variants are enriched in the vicinity of each other. We found a strong, positive correlation between SNV and deletion load at the gene level which is independent of ageing and genotype (Fig. 6b), indicating positional sensitivity to the accumulation of mutations which may reveal underlying genomic instability in specific regions or be caused by higher order structures.

## Discussion

Here we report a brain-wide spatio-temporal map of the mtDNA mutation spectrum in WT and proof-reading-deficient mice expressing *Polg*^*D181A*^ under the CaMKIIα-promoter. Using a PCR-free approach to enrich mtDNA for next-generation sequencing, we were able to study small tissue dissections while minimising bias to the analysis. Using this approach, we found that (1) the ageing-induced increase in SNVs and deletions is largely brain region-independent and reaches saturation at 50 weeks; (2) *Polg*^*D181A*^ expression specifically increase SNV and deletion levels in COR, NAc, and PVT; (3) ageing increases the number of shared SNVs, a feature that is enhanced in *Polg*^*D181A*^ mice and SNVs are prominent in the NCR; (4) deletions follow a bimodal size distribution independent of age and genotype; and (5) *Polg*^*D181A*^ induces NCR-containing multimers specifically in NAc and PVT in an ageing-dependent manner.

Deletions have been described in e.g. the brain [17, 30–34], muscle [35–37], and heart [30, 37]; however, all these studies suffer from limitations in either the amount of tissue required for input, a priori bias to the analysis, or limited temporal insight. Early studies tend to argue that deletions are mainly major arc deletions between the two origins of replication; however, we do not find any indication of specific accumulation of common mtDNA deletion between the origins of replication (Figs. 3b and 4a), similar to the mutator mouse [16]. Instead, we identified a highly diverse deletion spectrum ranging from 1 bp to 15 kb with a tendency for very short (< 10 bp) deletions to preferentially accumulate across all ages (Fig. 4b). The bimodal distribution of deletion lengths (Fig. 4b) was quite surprising. MtDNA deletions may be subject to two opposite working mechanisms: (1) shorter molecules, caused by larger deletions, finish replication faster, in principle leading to their rapid accumulation [23, 38]; (2) mtDNA undergo purifying selection [39], leading to the preferential loss of deleterious molecules (e.g. larger deletions) [40]. This bimodal distribution might be explained by the combination of these two mechanisms. However, the positive selection hypothesis has been disproved [41], and random genetic drift as the major contributor to the accumulation of short mtDNA species [42] was also recently questioned [43]. Furthermore, the presence of large deletions will naturally restrict the presence of smaller deletions. The description of a tight link between transcription and replication has increased interest in the positive selection idea [44], but the mechanisms remain unknown. However, the fact that young mice have a higher proportion of very small deletions (Fig. 4b) could favour a hypothesis including positive selection.

mtDNA deletions have been proposed to be prevalent between direct repeats [45, 46] in both normal ageing and disease, though this is not always the case [47, 48]. As such, deletions are grouped into type I (flanked by direct repeat) and type II (not flanked by direct repeat) [49]. Type I deletions are hypothesised to be formed by polymerase slippage during replication [50]. The discrepancy between human disease cases [51] and mouse models regarding direct repeats may relate to the different involvement of direct repeats in deletion formation between long-lived and short-lived mammals [26]. Type II deletions colocalize with 2D and 3D mtDNA structures [49] and occur spontaneously during replication either through strand slipping [48], *Polg* stalling [52], or repair of double-stranded breaks [53]. The fact that *Polg*^*D181A*^ expression increases deletion load without affecting the distribution of deletion sizes remains elusive. We speculate that *Polg*^*D181A*^-induced replication instability would increase the frequency of deletion-mediating events without effecting their overall properties or characteristics, but increased SNVs in itself do not seem to increase deletion rate [54].

Studies on duplications in mtDNA have previously focused on the D-loop [55–57], and we similarly found duplications to be a brain region- and NCR-specific ageing-induced *Polg*^*D181A*^-dependent event (Fig. 5a). NCR duplications could be caused by *Polg* stalling during replication [58]. Data indicates that the D-loop may not be an ideal region to use as a control in the estimation of mtDNA copy number [59], which could be related to the propensity of duplications in this region and may be partly due to direct repeats (Fig. 5d). In addition, the D-loop is often chosen for primer design for long-range PCR, why such long-range PCR is not suitable for rearrangement analysis without a priori knowledge of the multimer landscape which may be highly dependent on the animal model, the disease progression, or the tissue investigated. The D-loop has been proposed to directly interact with the inner mitochondrial membrane and through this interaction mediates protein recruitment and mitochondrial structure [12]. Multimers of NCR may therefore influence mitochondrial function in other ways than the classical view of energy production and mtDNA copy number.

We observed no differences in the types of SNV with ageing in neither WT nor *Polg*^*D181A*^ mice indicating that age-induced oxidative damage is not a major driver of mutations in mtDNA but rather mutations are caused by the accumulation of *Polg* errors in both WT and *Polg*^*D181A*^ mice. Oxidative damage of mtDNA causes 8-oxo-dG which was argued to result in G>T transversions [60]; however, we find that G>T only take up a small fraction of the identified SNVs, which could indicate that *Polg* is able to correctly incorporate T opposite 8-oxo-dG under oxidative conditions [61]. In support of this, oxidative damage is limited in mutator mice [6, 22, 62], data which can likely be extrapolated to our model. Instead, C>T and T>C are the major identified SNVs (Additional file 1: Fig. S1e). The major base interpretation mistake by *Polg* is T-GTP mispairing [63–66], though A>G can also occur due to deamination of adenosine. C>T is generally associated with cytosine deamination, though the exact link to *Polg* function is not clear. Overall, the mutation spectrum identified here is similar to that of the mutator mouse [19] as well as mtDNA analysis of ChIP-seq-derived data [24].

We found that SNVs tend to cluster in *hotspots* which display genotype and ageing characteristics (Fig. 2d). We found another *Polg*^*D181A*^-specific mtDNA mutation trait, the presence of multimers (Fig. 5a). The presence of several *Polg*^*D181A*^-dependent mutation traits compared to WT should caution the use of mtDNA mutation-inducing mouse models to describe ageing processes. We cannot testify to the tissue specificity of these observations, but at least the heart from mutator mice also seems to harbour genotype-specific multimers [16]. These mutations may not reflect the naturally occurring ageing phenotype adequately.

An unanswered question remains: what is the cause of regional sensitivity of proof-reading deficiency? As mitochondria are highly dynamic organelles that constantly undergo fission and fusion, the rates of these processes influence both replication and turnover of mtDNA. This is especially true in neurons, where mitochondria can roughly be divided into those found in the soma and those at the synapse with a constant transport of mitochondria along the axon [67, 68]. In addition, Parkin has been shown to protect SN neurons from the accumulation of mtDNA mutations in the mutator mouse [69], and it is likely that Parkin or other proteins influence similar processes in different brain regions. As the total level of *Polg* also appears to influence the propagation of deleterious mtDNA molecules [70], the transgenic expression of *Polg* may influence this regulation.

Elucidation of the cause and effect relationship between mtDNA mutations and ageing is not so straightforward. In mice, there is evidence that mtDNA SNVs themselves are adequate to induce premature ageing [71, 72], though the SNV load in these models is several fold higher than that observed in aged humans [73], and heterozygous *Polg*^*D257A*^ mice that also have elevated SNV levels do not show signs of premature ageing [6]. Human data suggest the ageing-dependent accumulation of mtDNA deletions [74], but whether this is a cause or consequence of ageing is not clear. Interestingly, mice lacking *Mgme1*, the mitochondrial exonuclease, accumulate deletions but do not show premature ageing [75], indicating the requirement of SNVs, not deletions, for premature ageing, likely by affecting the functionality of mitochondrial proteins or functional RNAs.

In all, our data provide a novel view of the spatio-temporal accumulation of mtDNA mutations by providing a method for investigating the full mutation spectrum from very limited tissue dissections. The differential response across brain regions to a state of replication instability provides insight into a possible heterogenic mitochondrial landscape across the brain that may help explain the specificity of neuropsychiatric disorders in individuals with mitochondrial disease as well as neurological changes associated with ageing. Appreciating the tissue and region specificities of the mitochondrial genome in terms of copy number variations [59, 76], mutations [14], or gene expression [77] is pivotal to understand changes in mitochondrial dynamics in ageing and disease states.

## Conclusions

We provide a novel unbiased spatio-temporal mapping of the full mtDNA mutation spectrum of discrete region-specific dissections of mouse brain using an approach that does not require PCR amplification of mtDNA prior to library preparation. We demonstrate that both single nucleotide variants (SNVs) and deletions accumulate homogeneously across the examined brain regions during ageing from 10 to 50 weeks but see no further increase towards 80 weeks. In mice expressing proof-reading-deficient *Polg*, *Polg*^*D181A*^, the mitochondrial response to this state of replication instability is highly brain region-specific, and we demonstrate that the paraventricular thalamic nucleus and nucleus accumbens are mutational *hotspots*. The increased mutation load in ageing *Polg*^*D181A*^ mice compared to wild-type is only moderately associated with changes in mutation characteristics. *Polg*^*D181A*^ also induces an ageing-dependent accumulation of non-coding control-region multimers, a feature that appears almost non-existent in wild-type mice. Our data show that unbiased sequencing of mtDNA from small tissue dissections can contribute to our understanding of the heterogenous mtDNA regulatory processes in ageing and disease states.

## Methods

### Animals

All animal care and experimental procedures were in accordance with the guidelines for proper conduct of animal experiments published by Science Council of Japan and approved by RIKEN Wako Animal Experiment Committee. All CaMKIIα-*Polg*^*D181A*^ transgenic mice used were heterozygotes. Animals were bred as described previously [14, 78]. In brief, mutant male CaMKIIα-*Polg*^*D181A*^ mice were mated with wild-type (WT) C57BL/6J female mice. Genotyping was performed using genomic DNA isolated from tail biopsies as described [14].

### Tissue sampling

Heterozygous female *Polg* mice (Tg (CaMKIIα-Polg^D181A^) C57BL/6J) and female WT littermates (10–11 weeks (10-week age group), 48–49 weeks (50-week age group), 81–84 weeks (80-week age group)) (*n* = 4–6 for each condition) were sacrificed by cervical dislocation, and the head was immediately removed and submerged in ice-cold modified ACSF (10 mM HEPES, 125 mM NaCl, 5 mM KCl, 2 mM CaCl_2_, 2 mM MgSO_4_, 10 mM glucose, pH 7.4). The whole brain was removed and washed in ice-cold ACSF and cut in 1-mm cortical sections using a brain matrix (ASI instruments, RBM-2000C). Areas of interest were identified and immediately dissected in ice-cold ACSF and snap-frozen in liquid nitrogen.

### mtDNA enrichment

Each sample was incubated at 37 °C for 16 h in 90 μL DNA buffer (10 mM TrisHCl pH 8.0, 0.1 M NaCl, 1% SDS) and 10 μL Proteinase K (Roche, #03115828001) and treated with RNase A for 10 min at room temperature. Total DNA was extracted using 1 vol AMPure XP beads (Beckman Coulter, #A63881). A small aliquot of the purified DNA (1/10 vol) was stored for qPCR and the remainder used for mtDNA enrichment. Total DNA was exonuclease treated for 36 h at 37 °C with interval shaking (1000 rpm) (18 μL total DNA, 3 μL NEBuffer 4 (10x), 4 μL ExoV (NEB, #M0345L), 6 μL ATP (10 mM)). Additional 1 μL NEBuffer 4 (10x), 2 μL ExoV, 6 μL ATP (10 mM) was added and incubated at 37 °C for 16 h. DNA was extracted using 0.4 vol AMPure XP beads. A small aliquot of the purified DNA (1/10 vol) was saved for qPCR. We note that the initial crude extraction of total DNA was unsuitable for the cerebellum due to the high lipid content of this tissue. For samples with high fibre density (e.g. NAc), we also periodically experienced some issues with solubility which was solved with either increased incubation time or increased buffer volume.

### qPCR validation of ExoV treatment

DNA stored before and after ExoV treatment was diluted and used to evaluate nDNA and mtDNA in the samples. qPCR conditions: 1 cycle (95 °C 30 s), 40 cycles (95 °C 5 s, 60 °C 30 s), 1 cycle (95 °C 15 s, 60 °C 60 s, 95 °C 15 s) (QuantStudio 12k Flex System, Applied Biosystems) using SYBR Premix Ex Taq (Takara, RR041) in 5-μL reaction volume. Primer sequences can be found in Additional file 2: Table S1.

### Library preparation and sequencing

Up to 0.5 ng DNA was prepared for sequencing using the NexteraXT DNA kit (Illumina, #FC-131-1024). Libraries were quantified using KAPA Universal kit (Kapabiosystems, #UKK4824) or Qubit dsDNA HS Assay (Invitrogen, #Q32851) and library size estimated by BioAnalyzer HS DNA chip (Agilent, #5067-4626). Libraries were pooled to 2 nM and sequenced on the Illumina MiSeq with 150-bp paired end reads (Illumina, #MS-102-2002). Note that the same number of PCR cycles for library amplification was used independent of start DNA input and that this did not lead to low complexity libraries or affected library size distribution as evaluated by BioAnalyzer.

### Sequencing analysis

Sequencing data was filtered, trimmed, mapped, and variant called using the *BBTools* suite (38.07) [21] using Clumpify (dedupe), FilterByTile (default), BBDuk (first: ktrim = r k = 23 mink = 11 hdist = 1 tbo tpe minlen = 100 ftm = 5 ordered (using adaptor resource provided with BBTools); second: k = 27 ordered qtrim = r trimq = 8 (using sequencing artefacts and phiX sequences provided with BBTools)), BBMap (vslow k = 11 secondary = t minratio = 0.55 tipsearch = 300 maxindel = 160000 ambig = all rescuedist = 30000 qtrim = lr), and CallVariants (rarity = 0.005 minallelefraction = 0.005 ploidy = 100 minedistmax = 5 border = 5 minquality = 10 minqualitymax = 10 minscore = 10) and postfiltered in R for minimum supporting reads (SNVs 4, deletions 2), sequencing depth (SNVs 50, deletions 25), and quality score (SNVs 20, deletions 10). Initially, reads were mapped to mm10 (ensembl) without the mitochondrial chromosome (MT) (using BBMap perfect mode) and unmapped reads were re-mapped to a modified version of MT consisting of two tandem MT sequences (in principle, a “double” MT chromosome, which we termed dMT) in order to call long-range deletions. We note that deletion calling is sensitive to kmer length chosen during mapping. Whereas a longer kmer showed more reproducible results, shorter kmers resulted in better detection sensitivity of deletions. Thus, we chose the shortest kmer value within the higher kmer lengths that gave reproducible results. The same number of aligned reads was used for each sample for variant analysis using random sampling with BBTools Reformat to avoid bias due to sequencing depth. Because of this approach, it is not necessary to normalise the read depth by the number of total reads. We filtered deletions to be a maximum of 15 kb based on our previous data indicating the presence of ~ 2 kb mtDNA molecules in the *Polg* mice [13]. For the analysis of multimers, only deletions with a length > 15 kb were retained, so no variants were included in both analyses.

SNV effect was evaluated using *SnpEff* [79]. Multi-sample overlap and statistics (Additional file 1: Figure S1C) was calculated using *SuperExactTest* [80]. Discordant reads were extracted using *samtools* (-F 1294).

The in silico deletion length-matched libraries were generated by randomly sampling *n* mtDNA positions (where *n* is the number of deletions in either WT or *Polg*^*D181A*^ samples), and these positions were used as deletion start positions. Deletion lengths (of either WT or *Polg*^*D181A*^ deletion libraries) were randomly shuffled and assigned to each randomly sampled mtDNA position, and the sum of these denoted the end position of the in silico generated deletions.

Statistical tests used are indicated in individual figure legends and were performed in *R* using base functions. To test the influence of ageing, genotype, animal, and/or region on the accumulation of mtDNA variants, we performed two- or three-way ANOVA. For pairwise testing, a two-sided *t* test (Welch’s) was performed. For pairwise testing of non-normal distributed data (as evaluated by Shapiro-Wilk’s method) that does not fulfil the central limits theorem, Wilcoxon testing was performed. All *p* values were Bonferroni corrected. Circular plots were made using the R package *circlize* [81].

To detect direct repeats in mtDNA, we split MT into 100-bp non-overlapping fragments and composed a local blast database (makeblastdb -in 100bp_fragments.fa -input_type fasta -dbtype nucl -out blastdb_100bp) against which we blasted the mtDNA sequence (blastn -task blastn-short -num_descriptions 500000000 num_alignments 500000000 –ungapped query mtDNA.fa -db blastdb_100bp -word_size 5 -evalue 1e300 –outfmt 6 –out mtblast). Putative direct repeats were post filtered to include maximum 1 mismatch per 4 nucleotides and for a minimum size of 8 bp due to the high frequency of shorter direct repeats.

Sequence identity scoring was performed using Needle (needleall in1.fa in2.fa -gapopen 10 -gapextend 0.5 -outfile out.txt) from EMBOSS [82].

### PCR and cloning

Total DNA was prepared from two separate littermate pairs (female) 35–37 weeks old as described above. PCR was performed using Tks Gflex (Takara, #R060A) with 5 ng total DNA/reaction. PCR was run using 1 cycle: 94 °C 2 min; 30 cycles: 94 °C 15 s, X°C 20 s, 68 °C Ys; 1 cycle: 68 °C 7 min, where X and Y were optimised for each primer set used. PCR products were run on 1% agarose gels in TAE buffer, and DNA was visualised using ethidium bromide. Primer sequences and primer-specific PCR conditions can be found in Additional file 2: Table S2. PCR products were purified from 1% agarose gels using Wizard SV Gel and PCR Clean-Up System (Promega), cloned (Invitrogen, #45-1641), and sequenced (BigDye Terminator v3.1, Applied Biosystems).

### RNA analysis

Tissues from 4 pairs of female *Polg* mice and littermate controls (10–12 weeks) were collected as described above but were immediately stored in TRIzol after dissection instead of snap-freezing. RNA was purified using the Direct-zol RNA Microprep Kit (Zymo Research, #R2060). One hundred nanograms of random hexamers (Invitrogen) was annealed (85 °C, 5 min) to 100 ng RNA. Reverse transcription was carried out with 100 U M-MLV Reverse Transcriptase (Invitrogen, #28025013) in a 13-μL volume supplemented with 10 mM DTT and 1 mM dNTP (NEB, #N0447S) at room temperature, 10 min, then at 37 °C, 60 min. cDNA was used for qPCR in technical triplicates. qPCR was performed as above. Primer sequences can be found in Additional file 2: Table S3.

## Supplementary information

**Additional file 1: Supplementary figures 1-5. Fig. S1:** Additional analysis of mtDNA single nucleotide variants. **Fig. S2:** Additional analysis of mtDNA deletions. **Fig. S3:** Additional analysis of mtDNA multimers. **Fig. S4:** Additional analysis of mtDNA multimers and inversions. **Fig. S5:** *Polg* expression analysis.

**Additional file 2: Supplementary tables 1-3. Table S1:** Table of primers used for qPCR and RT-qPCR to assess the levels of mtDNA, nDNA and mRNA targets. **Table S2:** Table of primers used for PCR to assess structural variants of mtDNA. **Table S3:** Table of primers used for qPCR to assess the expression of endogenous and transgenic *Polg*^*D181A*^.

## Data Availability

Sequencing data has been deposited to the Sequencing Read Archive at NCBI under accession number PRJNA623951. Analytical tools used in this study are cited within the “Methods” section.
