# Supplementary material for: Unbiased PCR-free spatio-temporal mapping of the mtDNA mutation spectrum reveals brain region-specific responses to replication instability
Source: BMC Biol. 2020 Oct 23;18:150. doi: 10.1186/s12915-020-00890-5 (PMC7585204; doi:10.1186/s12915-020-00890-5)
Supplement: Supplementary file 1 — Additional file 1: Supplementary figures 1-5. Fig. S1: Additional analysis of mtDNA single nucleotide variants. Fig. S2: Additional analysis of mtDNA deletions. Fig. S3: Additional analysis of mtDNA multimers. Fig. S4: Additional analysis of mtDNA multimers and inversions. Fig. S5: Polg expression analysis. [file 12915_2020_890_MOESM1_ESM.pdf]

Additional file 1: Unbiased PCR-free spatio-temporal mapping of the mtDNA mutation spectrum reveals brain region-specific responses to replication instability.

Emilie Kristine Bagge[1] (emilie.bagge@riken.jp), Noriko Fujimori-Tonou[1,2] (nfujimori@riken.jp), Mie Kubota-Sakashita[1] (mie.sakashita-kubota@riken.jp), Takaoki Kasahara[1,3] (takaoki.kasahara@riken.jp), Tadafumi Kato[1,4,*] (tadafumi.kato@juntendo.ac.jp)

[1] Laboratory for Molecular Dynamics of Mental Disorders, Center for Brain Science, RIKEN, Wako, Saitama, Japan

[2] Current address: Support Unit for Bio-Material Analysis, Research Resources Division, RIKEN Center for Brain Science, Wako, Saitama, Japan

[3] Current address: Career Development Program, Center for Brain Science, RIKEN, Wako, Saitama, Japan

[4] Department of Psychiatry and Behavioral Science, Juntendo University, Graduate School of Medicine, Tokyo, Japan

This file includes:

Fig. S1
Fig. S2
Fig. S3
Fig. S4
Fig. S5

Fig. S1

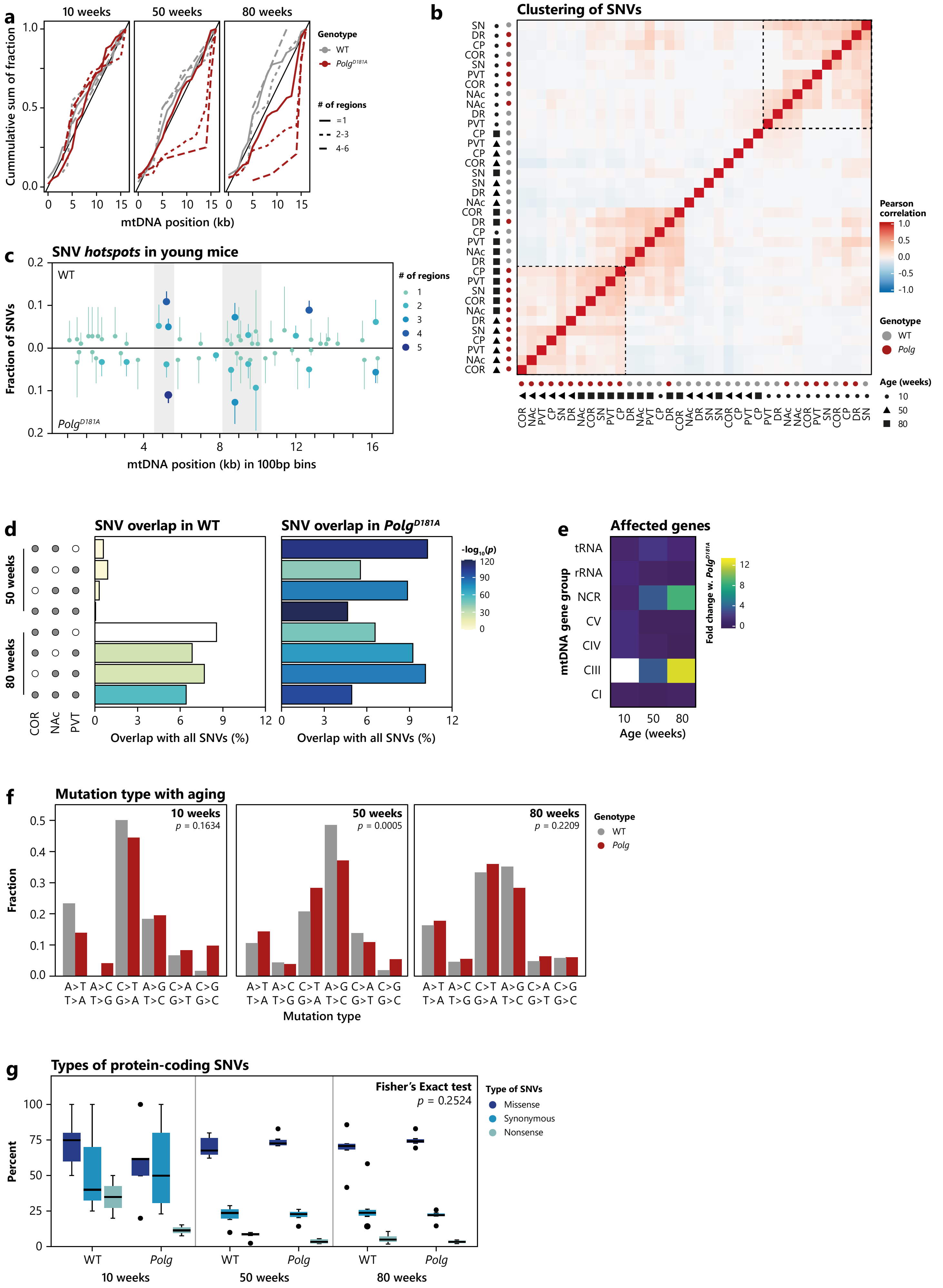
a
10 weeks
50 weeks
80 weeks
Cummulative sum of fraction
mtDNA position (kb)
Genotype
WT
Polg^D181A
# of regions
=1
2-3
4-6
b
Clustering of SNVs
Pearson correlation
Genotype
WT
Polg
Age (weeks)
10
50
80
c
SNV hotspots in young mice
WT
Polg^D181A
Fraction of SNVs
mtDNA position (kb) in 100bp bins
# of regions
d
SNV overlap in WT
SNV overlap in Polg^D181A
50 weeks
80 weeks
COR
NAc
PVT
Overlap with all SNVs (%)
-log10(p)
e
Affected genes
mtDNA gene group
tRNA
rRNA
NCR
CV
CIV
CIII
CI
Age (weeks)
Fold change w. Polg^D181A
f
Mutation type with aging
10 weeks
p = 0.1634
50 weeks
p = 0.0005
80 weeks
p = 0.2209
Fraction
Mutation type
Genotype
WT
Polg
g
Types of protein-coding SNVs
Fisher's Exact test
p = 0.2524
Percent
Type of SNVs
Missense
Synonymous
Nonsense
WT
Polg
10 weeks
50 weeks
80 weeks

Fig. S1

a SNVs were counted in 10bp non-overlapping bins for WT (grey) and *Polg*^{D181A} (red) mice at 10, 50, and 80 weeks and the number of regions with SNV in each bin calculated. Note that in the case that one region has more than one SNV in a bin, it is only counted as one instance of an SNV. The overlap was visualised for non-overlapping bins ("1"), bins shared across two or three regions ("2-3"), and bins shared across four to six regions ("4-6") as the percentual sum for each or these groups relative to mtDNA position.

b Pearson correlation of SNVs from all regions examined in both WT (grey) and *Polg*^{D181A} (red) samples for 10- (circle), 50- (triangle), and 80-week-old (square) mice.

c SNVs from 10-week-old WT (top) and *Polg*^{D181A} (bottom) were grouped into 100 bp bins and the mean fraction of SNVs in each bin per genotype is plotted. The number of brain regions contributing to SNVs in each bin is indicated by size and colour. Standard deviation for SNV fraction across brain regions is indicated.

d Overlap of SNVs at exact positions in mtDNA in COR, NAc, and PVT (filled circles indicates samples included in the comparison) from WT (left) *Polg*^{D181A}-mice (right) at 50 and 80 weeks. Bar height indicates percent of SNVs of total SNVs for each age. *p*-values are calculated independently for each age using *SuperExactTest* (with Bonferroni correction) and setting 16299 (the length of the mm10 mtDNA reference) as background.

e mtDNA-encoded genes were grouped by gene group and the fold change (FC) of the percentage of SNVs for the indicated age in WT animals (summed across all brain regions) with *Polg*^{D181A}-expression at each age is indicated.

f Types of mutations detected for WT (grey) and *Polg*^{D181A} (red) mice at 10 (left), 50 (middle) and 80 (right) weeks old expressed as the fraction of total SNVs. *p*-values indicate Fisher's Exact test for comparing the distribution across the 6 groups (i.e. mutation type) for WT and *Polg*^{D181A} at each age.

g All SNVs in protein-coding mtDNA genes are separated into *missense* (dark blue), *synonymous* (middle blue), and *nonsense* (light blue) for both WT and *Polg*^{D181A} at 10, 50, and 80 weeks old. Note that *Nonsense variants* include *start_lost*, *stop_gained* and variations of these. Fisher's Exact test for count data for comparing the distribution across the 3 groups (i.e. mutation group) for each sample type (i.e. genotype and age combination) are shown.

Fig. S2

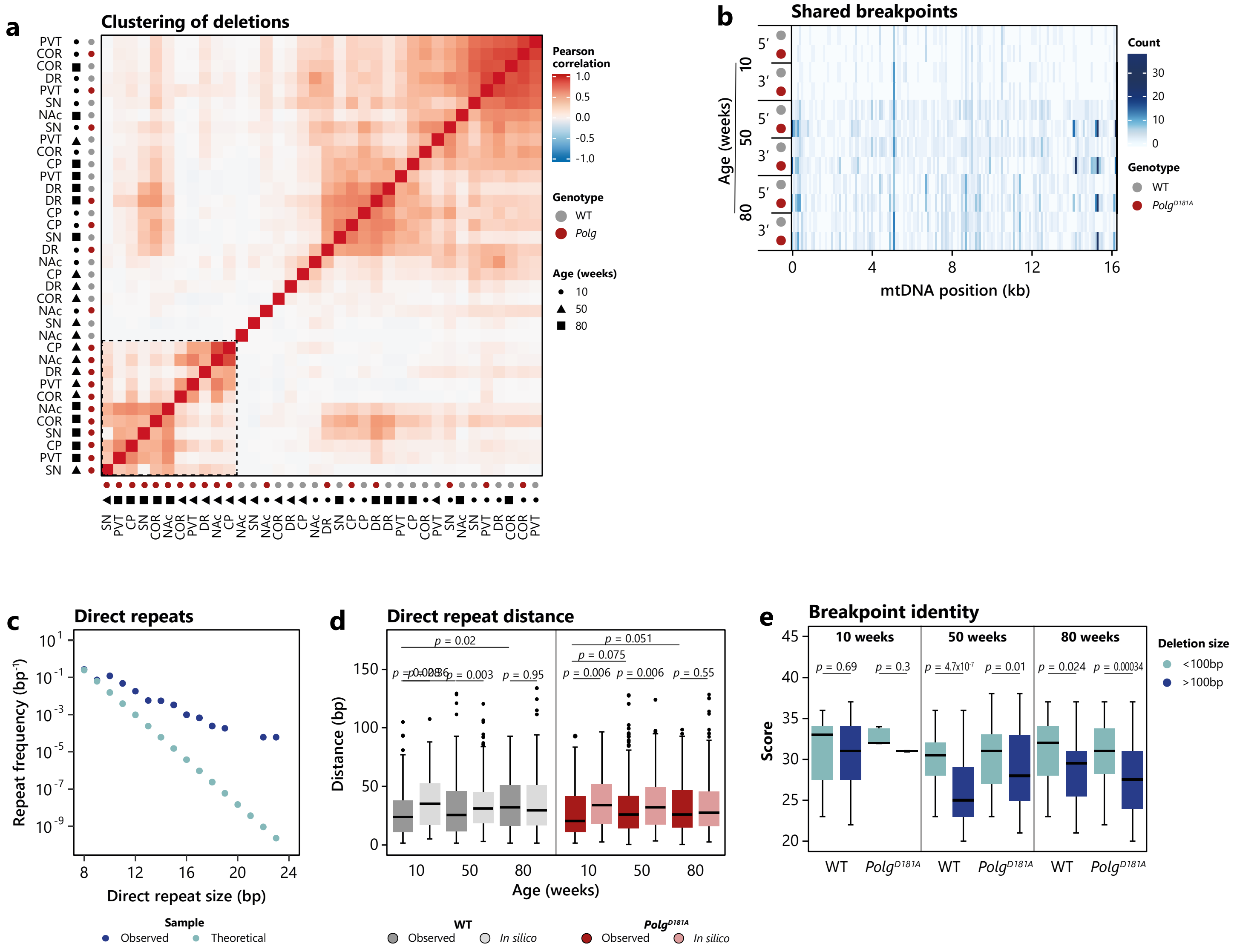

Fig. S2

a Pearson correlation of deletions from all regions examined in both WT (grey) and *Polg*^{D181A} (red) samples for 10- (circle), 50- (triangle), and 80-week-old (square) mice. Note the clear cluster containing *Polg*^{D181A} derived brain regions from both 50- and 80-week-old mice.

b Sharing of 5′ and 3′ deletion breakpoints were counted in non-overlapping 100 bp bins and shown in blue relative to mtDNA position at 10, 50, and 80 weeks for WT and *Polg*^{D181A} mice.

c The frequency of direct repeats as identified in the mitochondrial reference genome (dark blue) compared to the theoretical frequency based on the length of mtDNA (light blue) for the indicated direct repeat sizes.

d Distance of deletions to nearest direct repeat at each age for WT (grey) and *Polg*^{D181A} (red) as well as for in silico generated, length-matched deletion libraries (light grey and pink, respectively). *p*-values of two-sided *t*-tests are shown.

e *Needle* identity scores for WT and *Polg*^{D181A} separated by age and genotype and split into deletions <100 bp (light blue) and deletions >100 bp (dark blue). *p*-values of two-sided *Wilcoxon* tests are shown.

Fig. S3

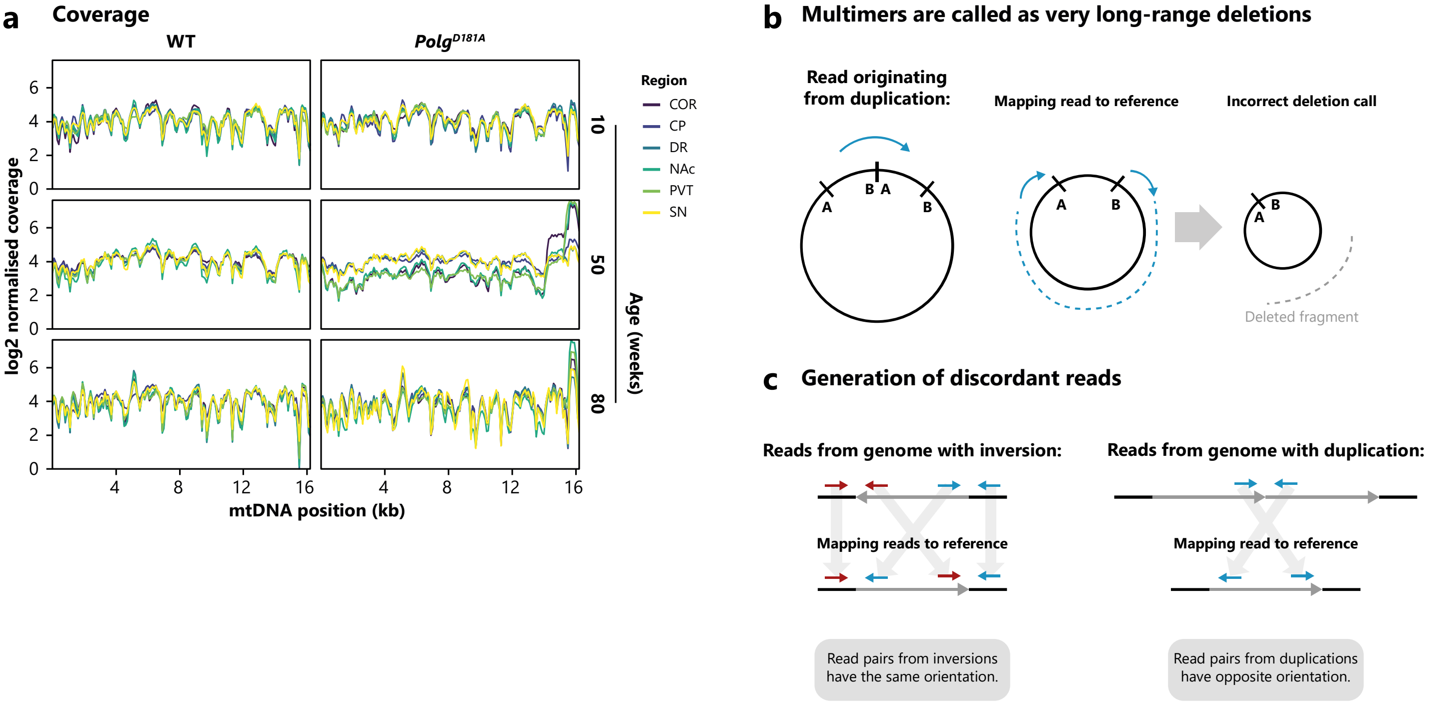

Fig. S3

a Coverage of the mitochondrial genome in WT (left) and *Polg*^{D181A} (right) for each region as indicated across ages.

b Illustration of how multimers can be incorrectly mapped as very long-range deletions. The read (in blue) originates from sequencing across the border of a multimer (first panel). During mapping, where split-read mapping is allowed, the read from this multimer is split (middle panel). During variant calling, the split read is interpreted as a very long-range deletion (final panel).

c Illustration of reads originating from genome rearrangements. (left) The origin of two read pairs (red and blue) are indicated on a DNA segment containing an inverted fragment (grey) where one read originates from the inverted fragment and one read originates from non-inverted DNA (i.e. DNA with expected orientation relative to the reference) (shown in red and blue). When mapping, the orientation of the inverted fragment is now in the reference orientation, leading to read pairs having the same orientation). (right) The origin of a read pair (in blue) are indicated on a multimeric stretch of DNA (grey) where one read originates from one copy and the other read originates from another copy of the multimer. When mapping, the orientation of the reads will be outward facing, due to only one copy of the multimeric fragment being present in the reference.

Fig. S4

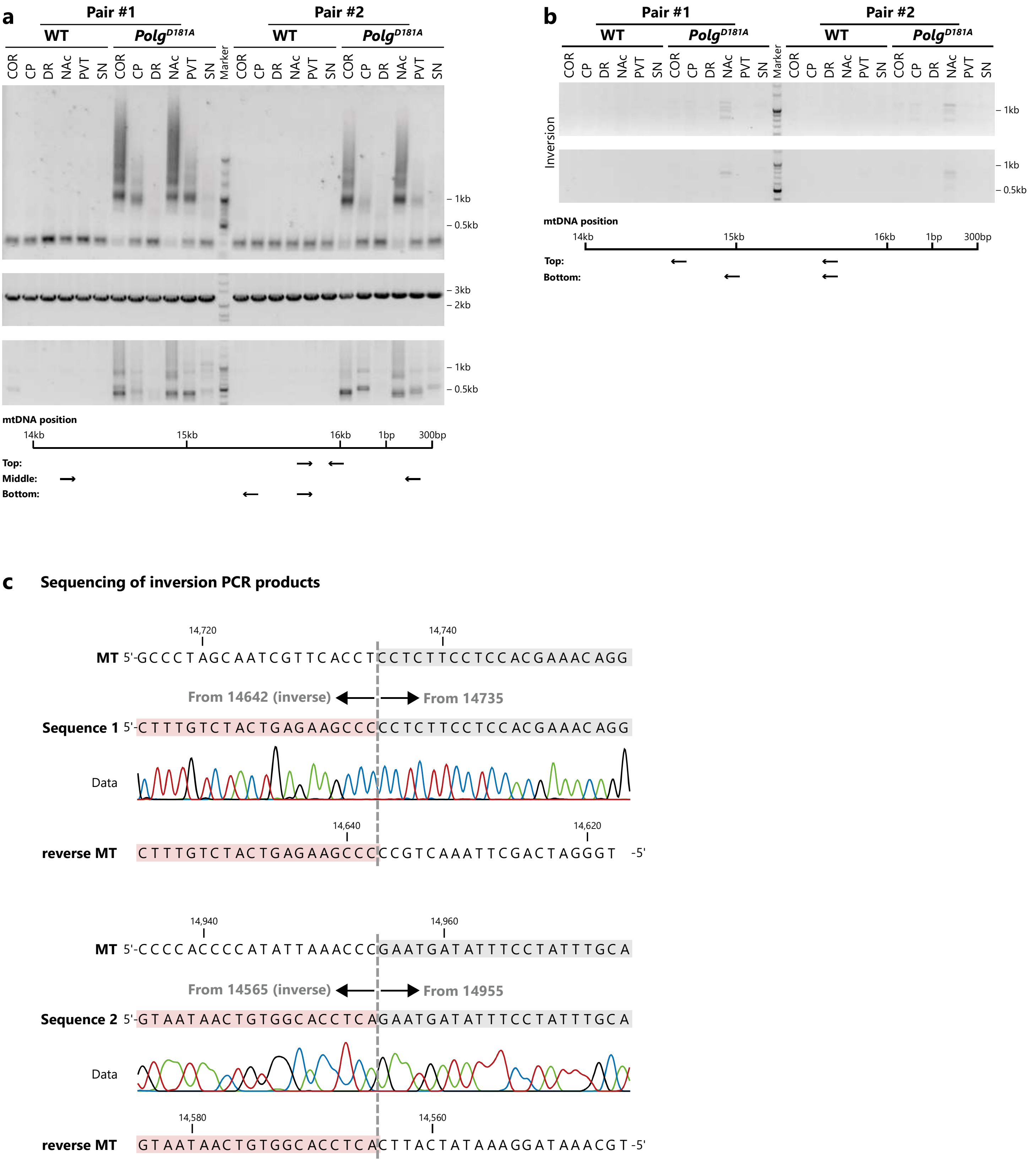
a
Pair #1
Pair #2
WT
Polg D181A
COR CP DR NAc PVT SN
Marker
1kb
0.5kb
3kb
2kb
mtDNA position
14kb
15kb
16kb
1bp
300bp
Top:
Middle:
Bottom:
b
Inversion
c
Sequencing of inversion PCR products
14,720
14,740
MT 5'-GCCCTAGCAATCGTTCACCTCCTCTTCCTCCACGAAACAGG
From 14642 (inverse)
From 14735
Sequence 1 5'-CTTTGTCTACTGAGAAGCCCCCTCTTCCTCCACGAAACAGG
Data
14,640
14,620
reverse MT CTTTGTCTACTGAGAAGCCCCCGTCAAATTCGACTAGGGT -5'
14,940
14,960
MT 5'-CCCCACCCCATATTAAACCCGAATGATATTTCCTATTTGCA
From 14565 (inverse)
From 14955
Sequence 2 5'-GTAATAACTGTGGCACCTCAGAATGATATTTCCTATTTGCA
Data
14,580
14,560
reverse MT GTAATAACTGTGGCACCTCACTTACTATAAAGGATAAACGT -5'

Fig. S4

a PCR results using primers within the putative duplication region (top and bottom) and outside (middle) using total DNA from the indicated brain regions in two unrelated littermate pairs of WT and *Polg*^{D181A} -mice. Primer position is shown below gel pictures.

b PCR results using two primer sets specific for inversion using total DNA from the indicated brain regions in two unrelated littermate pairs of WT and *Polg*^{D181A} mice. Primer positions are shown below gel pictures. For primer sequences and PCR details, see Methods.

c Sequence chromatograms of two sequences (1 and 2) of targets amplified by two different primer sets targeting inversions. DNA is derived from NAc, equivalent to that in Fig. S4A. mtDNA sequences matching the sequence is shown (in grey for sequence in correct orientation and pink for sequence in inverse orientation). For sequence 2, note the complementary sequence of the sequence 5′ to the "pink" sequence relative to the "grey" sequence.

Fig. S5

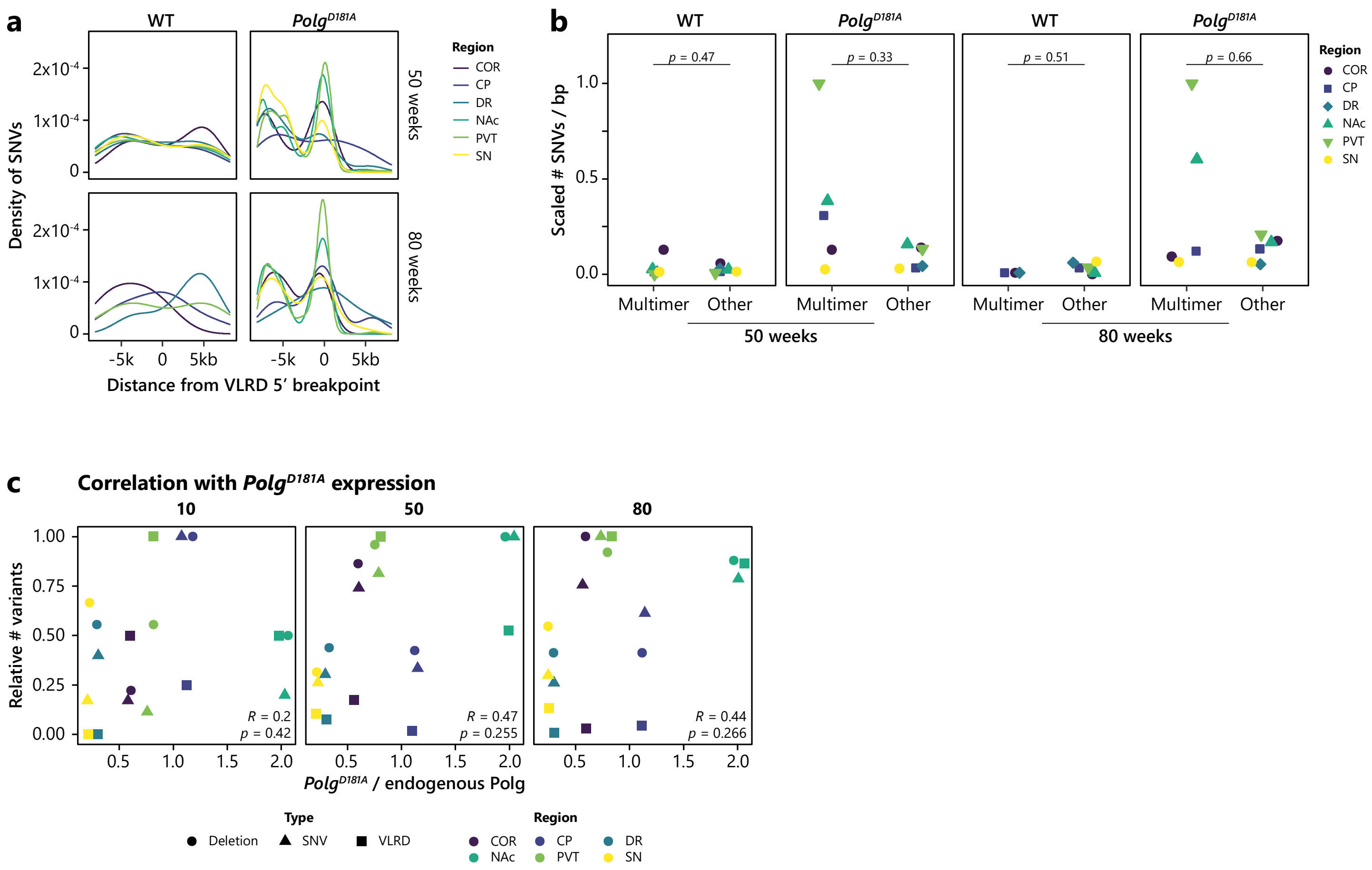

Fig. S5

a VLRD 5′ breakpoints were set as position 0 and the distance to all SNVs for all VLRDs was plotted as a density plot for each brain region for WT and PolgD181A mice.

b SNVs in a region encompassing the putative multimeric region (position 15.4kb to 15.8kb) was extracted and compared to SNVs outside of this region by plotting the scaled number of SNVs pr. bp. Two-way ANOVA (region, multimer) was performed for each age and *p*-values for *post-hoc* Tukey's test are shown.

c Scaled levels of the indicated types of variants shown as a function of the expression level of the *Polg*^{D181A} transgene relative to endogenous *Polg* for each region from *Polg*^{D181A}-mice as indicated by colour. Average expression levels are shown for n = 4.
