# Supplementary material for: Unbiased PCR-free spatio-temporal mapping of the mtDNA mutation spectrum reveals brain region-specific responses to replication instability
Source: BMC Biol. 2020 Oct 23;18:150. doi: 10.1186/s12915-020-00890-5 (PMC7585204; doi:10.1186/s12915-020-00890-5)
Supplement: Supplementary file 2 — Additional file 2: Supplementary tables 1-3. Table S1: Table of primers used for qPCR and RT-qPCR to assess the levels of mtDNA, nDNA and mRNA targets. Table S2: Table of primers used for PCR to assess structural variants of mtDNA. Table S3: Table of primers used for qPCR to assess the expression of endogenous and transgenic PolgD181A. [file 12915_2020_890_MOESM2_ESM.pdf]

Additional file 2: Unbiased PCR-free spatio-temporal mapping of the mtDNA mutation spectrum reveals brain region-specific responses to replication instability.

Emilie Kristine Bagge[1] (emilie.bagge@riken.jp), Noriko Fujimori-Tonou[1,2] (nfujimori@riken.jp), Mie Kubota-Sakashita[1] (mie.sakashita-kubota@riken.jp), Takaoki Kasahara[1,3] (takaoki.kasahara@riken.jp), Tadafumi Kato[1,4,*] (tadafumi.kato@juntendo.ac.jp)

[1] Laboratory for Molecular Dynamics of Mental Disorders, Center for Brain Science, RIKEN, Wako, Saitama, Japan

[2] Current address: Support Unit for Bio-Material Analysis, Research Resources Division, RIKEN Center for Brain Science, Wako, Saitama, Japan

[3] Current address: Career Development Program, Center for Brain Science, RIKEN, Wako, Saitama, Japan

[4] Department of Psychiatry and Behavioral Science, Juntendo University, Graduate School of Medicine, Tokyo, Japan

This file includes:

Table S1

Table S2

Table S3

Table S1.

Table of primers used for qPCR and RT-qPCR to assess the levels of mtDNA, nDNA and mRNA targets.

Target	Forward (5'-3')	Reverse (5'-3')
**mtDNA1**	AATTCAAGCCTACGTATTCACC	AGGGTTAATAGTGTAATTGAAT
**mtDNA2**	AACCGAGTCGTTCTGCCAATAG	TTCTAGGACAATGGGCATAAAG
**mtDNA3**	TTACATCTGTAGCCCTTTTTGT	TCGTCCGTACCATCATCCAATT
**nDNA1 (*mKlf4*)**	TCCTTAGGGCTTTCTTGGGT	GCACATTCAGGCAGCAGATA
**nDNA2 (*mGapdh*)**	ATGGGTGTGAACCACGAGAA	TCCACTCATGGCAGGGTAAG
**nDNA3 (*mMyc*)**	CTCGCCCAAATCCTGTACCT	TCCTACCCTGCTGTGAATGG

Table S2.

Table of primers used for PCR to assess structural variants of mtDNA. “Rx” denotes the primer specific annealing temperature and elongation time.

Target	Sequence (5’ to 3’)	Sequence (5’ to 3’)	Rx	Figure
**D-loop**	CACCAATGCCCCTCTTCTCG (F)	TTGGGTTTTGCGGACTAATGAT (R)	50°C 120s	S4A Top
**3’-region**	TAAAATTATTAACCACTCAT (F)	TGTGGCTAGGCAAGGTGTCT (R)	50°C 120s	S4A Middle
**Duplica-tion**	CACCAATGCCCCTCTTCTCG (F)	CATAGTACAACAGTACATTTATGTATATCG (R)	57°C 45s	S4A Bottom
**Inversion**	AAGGGGATAGTCATATGGAAG (R)	GGTGGCTTTGTCTACTGAGA (R)	57°C 60s	S4B Top
**Inversion**	AAGGGGATAGTCATATGGAAG (R)	AGGACACCTCCTAGTTTATTG (R)	55°C 60s	S4B Bottom

Table S3.

Table of primers used for qPCR to assess the expression of endogenous and transgenic *Polg*^{D181A}.

Target	Forward (5’-3’)	Reverse (5’-3’)
**mut*Polg1***	GGCATGACTTCTGCGCTAA	GATTGTCTTTTCTGACCAGATGG
**Wild type *Polg1***	AGCTTGACTGCTTTTAGTGGC	GCCGCAGAACGGGAAG
Gapdh	ATGGGTGTGAACCACGAGAA	TCCACTCATGGCAGGGTAAG
